# Emotional Disorders, Risk Factors, and Correlations of Post-Partum Depression and Post-Traumatic Stress Disorder with Sexual Function During Post-Partum Period

**DOI:** 10.3390/diagnostics15091065

**Published:** 2025-04-22

**Authors:** Panagiotis Eskitzis, Vasiliki Michou, Christiana Arampatzi, Ioannis Tsakiridis, Dimitrios Papoutsis

**Affiliations:** 1 Department of Midwifery, School of Healthcare Sciences, University of Western Macedonia, Keptse, 50200 Ptolemaida, Greece; peskitzis@uowm.gr (P.E.); xristianaarampatzi@gmail.com (C.A.); dpapoutsis@uowm.gr (D.P.); 2 3rd Department of Obstetrics and Gynecology, Aristotle University of Thessaloniki, Ippokrateio General Hospital of Thessaloniki, Kostantinoupoleos Street 49, 54642 Thessaloníki, Greece; igtsakir@auth.gr

**Keywords:** sexual health, puerperium, post-partum depression, post-traumatic stress

## Abstract

**Background:** This study aimed to investigate the presence of emotional disorders, the risk factors associated with these disorders, and the level of sexual function observed after childbirth. Additionally, the study aimed to explore how sexual function affects post-partum depression and Post-Traumatic Stress Disorder (PTSD), as well as how these emotional disorders, in turn, impact sexual function. **Methodology:** A total of 336 women participated in the study, who were asked to complete four questionnaires: a general women’s personal information questionnaire, the Edinburgh Postnatal Depression Scale (EPDS), the PTSD Scale (PCL-5), and the Female Sexual Functioning Index (FSFI). **Results:** The results showed that 33% of mothers scored on the EPDS above 14 points, which was considered a threshold value for the prognosis of post-partum depression. In addition, the women scored an average of 20.8 points on the FSFI, and thus, their level of sexual functioning was characterized as moderate. According to the total score of the PCL-5 scale, it was observed that 17.6% of the mothers show post-traumatic stress after childbirth and satisfy all four criteria of this scale. Lastly, multiple regression analysis showed that factors such as annual family income and negative body image had a significant contribution to the models. **Conclusions:** Finally, it was observed that reduced sexual functionality in women is linked to post-partum depression and post-traumatic stress after childbirth. In conclusion, our research emphasizes the need for further exploration of the psychological and emotional challenges women face during the puerperium, which can negatively affect sexual health.

## 1. Introduction

The puerperium, also known as the post-partum period, is the phase following childbirth when a woman’s body gradually returns to its pre-pregnancy state. Often referred to as the “fourth trimester” of pregnancy by some researchers, this period begins with the birth of the child and the delivery of the placenta. While it typically lasts between four to six weeks, some older literature suggests it may extend up to eight weeks. It is important to recognize that the duration of the post-partum period can vary for each woman. Significant physiological and anatomical changes occur during this time, which can lead to discomfort and physical tension [1]. Moreover, the duration of pregnancy, childbirth, the post-partum period, and breastfeeding can significantly impact a woman’s physiological and psychological functions, which may adversely affect her sexuality.

Sexuality is a fascinating and complex process that encompasses not only gender but also sexual identity and orientation, as well as eroticism, pleasure from physical contact, and intimacy [2]. Sexuality is shaped by various factors, including biological, psychological, social, economic, political, cultural, ethical, legal, historical, religious, and spiritual influences. Sexuality is a multidimensional phenomenon that refers to integrating individual life experiences. It has also been described as a subjective experience that varies from individual to individual, often involving both the person and their partner at physical, emotional, and psychological levels. In the case of women, sexuality is particularly influenced by experiences and events related to pregnancy, childbirth, and motherhood, which can have a profound impact on their physical and emotional well-being. Especially regarding sexual functionality after childbirth and during the puerperium, age, changes in the physiology of the organs, body image, hormonal environment, and support from the partner and the wider family environment can be considered important factors, as they exert a great influence on the sexuality of post-partum women [3,4,5].

Post-partum depression and sexual dysfunction are common and interrelated complications that can significantly impair maternal mental health, interpersonal relationships, and quality of life [4]. Beyond psychosocial and environmental factors, recent evidence highlights a neurobiological basis involving the dysregulation of the hypothalamic–pituitary–adrenal (HPA) axis and disruptions in neurosteroid signaling—particularly involving allopregnanolone, a GABA_A receptor modulator crucial for mood, anxiety, and sexual behavior regulation. The abrupt decline in allopregnanolone post-partum is implicated in both depressive symptoms and sexual dysfunction. Emerging research supports the neuroprotective role of allopregnanolone in mitigating neuroinflammatory and neurotoxic stressors. For instance, Mohammed et al. [6] and Salahuddin et al. [7] demonstrated its potential in restoring neuroendocrine balance under conditions of steroidogenesis disruption and neuroinflammation. These findings underscore the importance of hormonal homeostasis in post-partum mental and sexual health. Globally, post-partum depression affects approximately 10–15% of new mothers, with symptoms typically emerging within the first 4–6 weeks post-partum [8], though onset may occur up to a year after childbirth [9,10,11]. Risk factors include prior mental health disorders, perinatal stressors (e.g., preterm birth), and insufficient social support [12,13]. Importantly, PPD is associated with sexual dysfunction in both mothers and their partners, with studies reporting concern rates of 33–67% in mothers and 33–77% in partners [14,15]. Recent findings support a strong positive correlation between post-partum depressive symptoms and sexual dysfunction [16].

Similarly, Post-Traumatic Stress Disorder (PTSD) has a significant impact on the mental health of post-partum women and can also lead to sexual problems during the post-partum period. A traumatic birth experience (i.e., an unplanned emergency cesarean section or a long, painful labor) in a woman’s life can act as a significant stressor and trigger the development of PTSD [17]. This mental illness disproportionately affects women of reproductive age [18]. There is also evidence indicating that this trauma can influence various aspects of a couple’s relationship, including sexual functioning, desire for intercourse, and intimacy [19]. It is also crucial to emphasize that cohort studies assessing sexual functioning in women during the post-partum period, particularly regarding post-partum depression and PTSD, are rarely conducted or face challenges in gaining acceptance from the intended participants in Greece. Therefore, the need for more cross-sectional and intervention studies and cohort studies in our country is not just important, but urgent. Thus, this study aimed to examine the relationship between sexual function and emotional disorders following childbirth. We hypothesized that sexual function is correlated with post-partum depression and PTSD, with each influencing the other. Additionally, considering that several risk factors can significantly affect the emotional well-being of post-partum women, potentially leading to post-partum depression and/or PTSD, we proposed that factors such as age, family income, marital status, support from family or partners, history of emotional disorders, type of delivery, breastfeeding practices, body image, and whether the infant was born full-term or pre-term are associated with post-partum depression and PTSD.

## 2. Materials and Methods

### 2.1. Recruitment

Post-partum women and new mothers were recruited through online websites and motherhood forums. Those who voluntarily expressed interest in participating were screened for eligibility based on specific inclusion and exclusion criteria. The inclusion criteria included being over 18 years of age and having given birth within the past 12 months. The exclusion criteria included being under 18 years old, prior participation in a similar study, and refusal to provide the necessary information to the researchers.

### 2.2. Study Design

This research was approved on 14 February 2023 by the Research Ethics Committee of the Department of Medicine at Aristotle University of Thessaloniki (AUTH), Greece, with protocol number 89/2023. An invitation to voluntarily participate in the study was shared with post-partum women and new mothers through various websites and forums focused on motherhood. Post-partum women received detailed information about the study’s research protocol, including its objectives, methodological approach, confidentiality measures, and adherence to the General Data Protection Regulation (GDPR) under European Union Regulation 2016/679. Those interested in participating were required to complete a consent form. Then, they were asked to fill in four questionnaires: a questionnaire regarding demographic and other personal information, the Edinburgh Postnatal Depression Scale (EPDS), the Post-traumatic Stress Checklist, PCL-5, and the Female Sexual Function Index (FSFI). This study involves the results of FSFI that were previously published in a relevant article that evaluated the FSFI in post-partum women and its correlation with sexual function risk factors [20].

### 2.3. Sample Size Calculation

The sample size for this study was calculated using the formula for estimating a mean in a single population. According to a study by Al-Garni et al. [21], the mean score for post-partum depression, measured using the EPDS, was 9.4 ± 0.7. Additionally, the estimated prevalence of post-partum depression was reported as 28% (*p* = 0.28). A 95% confidence level (Z = 1.96) and a desired margin of error (d) of 0.05 were used as parameters in the calculation. Based on these inputs, the required sample size was estimated to be approximately 339 participants. However, when calculated using the prevalence estimate and the same level of precision, a minimum of 310 participants was found to be sufficient. The final sample size was determined by integrating both the estimated mean and prevalence to ensure adequate statistical power and representativeness.

### 2.4. General Questions Regarding Demographic and Other Personal Information

The participants were asked to complete a questionnaire developed by the researchers. This questionnaire gathered demographic and relevant information, including gender identity, age, sexual orientation, smoking habits, alcohol consumption, body image, educational background, annual family income, employment status, and marital status. Additionally, the participants provided information about the support they receive from their spouse or partner and insights into their overall family environment. The questionnaire also included questions about childbirth, addressing aspects such as the type and timing of the experience, along with other key details.

### 2.5. Edinburgh Postnatal Depression Scale

EPDS is a valid, reliable, and widely used 10-item scale that is easy to complete for screening symptoms of postnatal depression in both the early and late perinatal period. Each question comprises four possible answers that reflect how the woman has felt over the past week rather than just her feelings when completing each EPDS question. The answers to questions 1, 2, and 4 are scored with 0, 1, 2, and 3 points based on the severity of the symptoms. Conversely, the answers to questions 3, 5, 6, 7, 8, 9, and 10 are scored inversely to the above questions (i.e., with 3, 2, 1, and 0 points, respectively). The total score is the sum of the scores for all the questions. When the resulting score exceeds 12 or 13 points, it indicates that mothers may be experiencing post-partum depression and should seek medical assistance [22]. The EPDS has been translated, culturally adapted, and psychometrically validated across diverse countries and populations. In the present study, we utilized the Greek version of the EPDS, which was translated and validated by Leonardou et al. [23] in a cohort of 81 Greek mothers at two months post-partum. The translation and cultural adaptation process adhered to the “Translation and Cultural Adaptation of Patient-Reported Outcomes Measures—Principles of Good Practice” guidelines [24]. The adapted version of the questionnaire was administered to 81 post-partum women at the University Clinic of Women’s Mental Health, National and Kapodistrian University of Athens. The findings confirmed that the Greek version of the EPDS is a valid and reliable instrument for the detection of post-partum depression, demonstrating robust psychometric properties. Specifically, the analysis identified an optimal cut-off score of 11/12 for the identification of post-partum depression, with a sensitivity of 90% and a specificity of 97.2% [23,24].

### 2.6. Post-Traumatic Stress Checklist

PCL-5 is a widely used 20-item self-report scale that assesses symptoms of post-traumatic stress in the past month according to 4 criteria (B, C, D, and E), as defined in the fifth edition of the *Diagnostic and Statistical Manual of Mental Disorders* (DSM-5) [25]. The severity of post-traumatic stress symptoms is assessed by summing the scores from 20 individual questions. Each question offers five possible responses on a Likert-type scale. The answers are scored as follows: 1 (not at all), 2 (a little), 3 (moderately), 4 (quite a lot), and 5 (extremely) [26,27]. A potential diagnosis of PTSD can be considered if the following conditions are met:

- Each item’s score equals or exceeds 2 (with a scoring range of 0 to 4).
- The total score for criteria B (avoidance) and C (negative alterations in cognition and mood) equals or exceeds 1.
- The total score for criteria D (alterations in arousal and reactivity) and E (duration of symptoms) is equal to or greater than 2.

Additionally, the scores from each question on the PCL-5 (with a total range of 0 to 80) should also be summed [25,26,27]. Additionally, regarding the weighting and reliability of the PCL-5 questionnaire, studies have shown that Cronbach’s alpha index ranges from 0.76 to 0.97 [28,29,30]. In a recent study assessing post-traumatic stress symptoms in Greek mothers following cesarean sections, the validation and weighting of the PCL-5 scale were examined. The results indicated that the PCL-5 scale had high reliability, with a Cronbach’s alpha coefficient of 0.97. Additionally, the scale demonstrated strong validity, as indicated by individual correlations based on Spearman’s statistical protocol, which ranged from 0.443 to 0.835. These findings suggest a significant positive correlation between various types of traumatic events and the scores on the PCL-5 scale [30].

### 2.7. Female Sexual Functioning Index

FSFI is a user-friendly tool for assessing six aspects of female sexual function: desire, arousal, lubrication, orgasm, satisfaction, and pain. It consists of 19 multiple-choice questions scored using a 5-point Likert scale, each with a multiplying factor between 0.3 and 0.6. Each domain has a maximum score of 6, resulting in a total possible score of 36. The minimum score for each domain ranges from 0 to 1.2, leading to an overall minimum FSFI score of 2 points [31]. Research indicates that a total FSFI score of 26 points or lower reflects the presence of sexual dysfunction more accurately (specificity = 0.733; sensitivity = 0.889). It is also advisable to consider the individual scores for each domain, both within the model and conceptually, to improve the accuracy of differential diagnoses among the various types of female sexual dysfunction [32]. In a previous study conducted by our team regarding the FSFI evaluation and its correlation with sexual function risk factors, as well as in this study, the Greek version of the FSFI was utilized [20]. This development involved five native Greek speakers, including a psychologist, a urologist, a gynecologist, and two translators, who translated the English FSFI to ensure accuracy. The translations were merged into a single version and then backtranslated into English for comparison with the original. The new Greek version was tested with five individuals to assess comprehension and cultural relevance. Following this evaluation, the final version was prepared for statistical validation [33]. The Greek FSFI showed a high Cronbach’s alpha reliability of 0.92, comparable to studies by Rosen et al. [31] (0.88) and Rillon et al. [34] (0.95), which reported a range of 0.89 to 0.97.

### 2.8. Statistical Analysis

Statistical analyses were performed using IBM SPSS Statistics for Windows, Version 28.0 (IBM Corp., Armonk, NY, USA). Descriptive statistics were initially used to summarize the data, including frequencies (n) and percentages (%) for categorical variables. Continuous variables, including subscale scores from the EPDS, PCL-5, and FSFI, were reported as means (M) ± standard deviations (SD), with the minimum (Min) and maximum (Max) values where relevant. No missing data were observed for the EPDS, PCL-5, or FSFI in the analyzed dataset. The internal consistency reliability of each scale was evaluated using Cronbach’s alpha coefficients, with values ≥0.70 considered indicative of acceptable reliability. To examine the influence of potential confounding variables—including age, family income, marital status, social support (from family or partner), history of emotional disorders, delivery type, breastfeeding practices, and body image—on the EPDS and PCL-5 total scores, multiple linear regression analyses were conducted. Statistical significance was set at *p* < 0.05 for all the analyses.

## 3. Results

### 3.1. Participants’ Personal and Demographic Data

A total of 336 heterosexual post-partum women voluntarily consented to take part in this study. Most participants were between 31 and 35 years old (36.9%). A significant number were married (93.2%) and did not engage in any form of exercise (59.8%). Most had a moderately positive body image (40.5%) and received strong supportive care from their partner (67.9%) and family (63.4%) following childbirth. The majority experienced a normal delivery (36.0%) and had a full-term infant (78.9%). Additionally, many reported a positive birth experience that met their expectations (71.7%). At the time of the study, 43.8% were still breastfeeding, and 69.6% had not experienced any emotional disorders in the past. Furthermore, 67.9% did not have a family history of emotional disorders, including depression, anxiety disorders, or bipolar disorder (see Supplementary Tables S1–S4) [20].

### 3.2. EPDS Results

**Figure 1:**
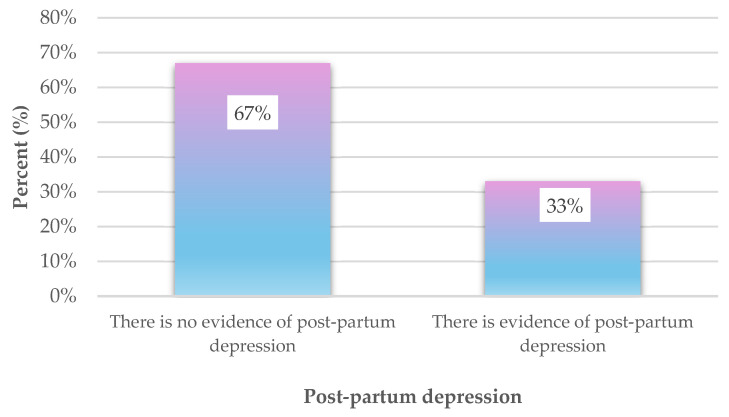
Percentage of women in the research sample with signs of post-partum depression.

Table 1 presents the findings from the descriptive analysis of the 10 questions on the EPDS, which assesses symptoms of post-partum depression. The results indicate that the most frequently reported symptom among women was feeling stressed or anxious for no specific reason (M = 1.8, SD = 1.0). This was followed by difficulties meeting obligations (M = 1.4, SD = 0.8) and self-blame for issues without reason (M = 1.2, SD = 0.9). Overall, the data suggest that women, on average, rarely experienced the symptoms measured by the EPDS, with mean values ranging from 0.5 to 1.8 on a scale that ranges from 0 to 3 (Table 1). The results indicated that the average score on the EPDS for the research sample was 10.2, with a standard deviation of 6.2 (Cronbach’s alpha = 0.876). The scores ranged from 0 to 27. Additionally, it was observed that 33% of the women in the study sample, totaling 111 participants, exhibited signs of post-partum depression (Figure 1).

### 3.3. Post-Partum Depression Risk Factors

A multiple regression analysis revealed the relationship between the total score of the EPDS and various independent variables. The results showed that annual family income (*p* = 0.025), body image (*p* < 0.001), partner (*p* = 0.001) and family (*p* = 0.024) support, birth expectations (*p* = 0.030), presence of intense traumatic experience in the perinatal period (*p* < 0.001), and presence of personal emotional disorder in the past (*p* < 0.001) had a significant contribution to the model (Table 2). To be more precise, the results revealed that 36.2% of the variability observed in the total score of EPDS was explained by the regression model (R^2^ = 0.362, F = 7.694, *p* < 0.001).

### 3.4. PCL-5 Results

**Figure 2:**
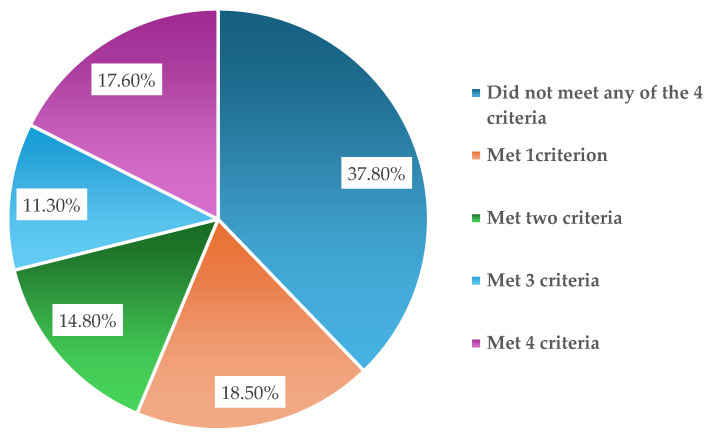
Results for the criteria related to post-traumatic stress disorder among all the women in the study’s sample.

**Figure 3:**
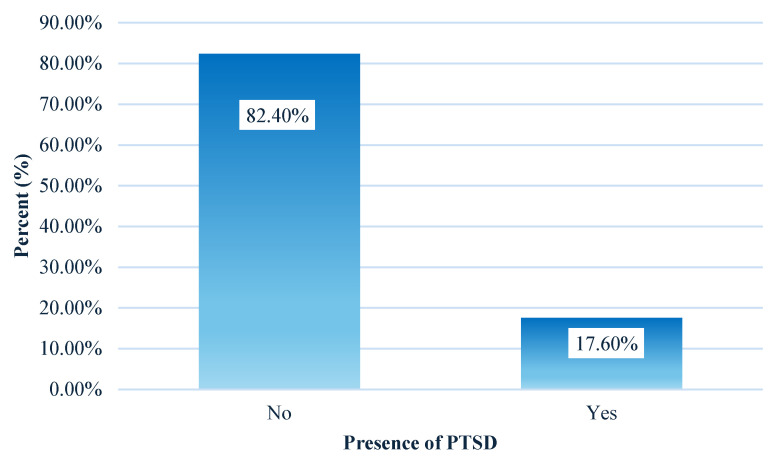
Percentage of women in the research sample with PTSD.

The descriptive results for the 20 questions on the PCL scale are presented in Table 3. The findings indicate that the mean values for most PCL scale questions fall between 0 (not at all) and 1 (a little), with some questions showing a mean value that is slightly above 1 (a little). Overall, it appears that the symptoms associated with post-traumatic stress disorder are generally low in frequency. Moreover, Figure 2 illustrates the results for the criteria associated with PTSD that all the women in the sample were evaluated against. The findings indicate that 37.8% of the women did not meet any of the four criteria for PTSD. In contrast, 18.5% met one criterion, 14.80% met two criteria, 11.3% met three, and 17.6% met all four criteria. Regarding the results for the percentage of women meeting the criteria for each of the four criteria for post-traumatic stress disorder, the results showed that 43.2% (*n* = 145) of the women met criterion “D”, 41.7% (*n* = 140) of the women met criterion “E”, 34.2% (*n* = 115) of the women met criterion “B”, and 33.3% (*n* = 112) of the women met criterion “C” (Table 4). Thus, based on the results of the PCL-5 scale, the results show that a total of 17.6% (*n* = 59) of the post-partum women experience post-traumatic stress after childbirth (Figure 3). In addition, this study showed that the PCL-5 demonstrated good internal consistency in the post-partum women (Cronbach’s alpha = 0.936).

The descriptive results for the 20 questions on the PCL scale are presented in Table 3. The findings indicate that the mean values for most PCL scale questions fall between 0 (not at all) and 1 (a little), with some questions showing a mean value that is slightly above 1 (a little). Overall, it appears that the symptoms associated with post-traumatic stress disorder are generally low in frequency. Moreover, Figure 2 illustrates the results for the criteria associated with PTSD that all the women in the sample were evaluated against. The findings indicate that 37.8% of the women did not meet any of the four criteria for PTSD. In contrast, 18.5% met one criterion, 14.80% met two criteria, 11.3% met three, and 17.6% met all four criteria. Regarding the results for the percentage of women meeting the criteria for each of the four criteria for post-traumatic stress disorder, the results showed that 43.2% (*n* = 145) of the women met criterion “D”, 41.7% (*n* = 140) of the women met criterion “E”, 34.2% (*n* = 115) of the women met criterion “B”, and 33.3% (*n* = 112) of the women met criterion “C” (Table 4). Thus, based on the results of the PCL-5 scale, the results show that a total of 17.6% (*n* = 59) of the post-partum women experience post-traumatic stress after childbirth (Figure 3). In addition, this study showed that the PCL-5 demonstrated good internal consistency in the post-partum women (Cronbach’s alpha = 0.936).

### 3.5. PTSD Risk Factors

The multiple regression analysis revealed the relationship between the total score of PTSD, based on the PCL-5 and various independent variables. The results showed that annual family income (*p* = 0.024), body image (*p* < 0.001), partner (*p* = 0.007) and family (*p* = 0.005) support, term of baby born (*p* < 0.001), type of delivery (*p* = 0.046), birth expectations (*p* < 0.001), presence of intense traumatic experience in the perinatal period (*p* < 0.001), and presence of personal emotional disorder in the past (*p* < 0.001) had a significant contribution to the model (Table 5). To be more precise, the results revealed that 39.9% of the variability observed in the total score of PTSD, based on the PCL-5, was explained by the regression model (R^2^ = 0.399, F = 8.995, *p* < 0.001).

### 3.6. FSFI Results

Table 6 shows the descriptive statistics for the six subscales and the total score of the FSFI scale. The results indicate that women in the post-partum period experienced moderate sexual functioning, with a mean score of 20.8 and a standard deviation of 11.3. Among the different dimensions of sexual functioning, the lowest scores were observed in arousal (mean = 3.1, SD = 2.2) and orgasm (mean = 3.2, SD = 2.4). In contrast, the highest scores were found in satisfaction (mean = 3.9, SD = 1.9) and pain (mean = 3.6, SD = 2.4) [20]. Lastly, this study showed that the FSFI demonstrated good internal consistency in post-partum women (Cronbach’s alpha = 0.944).

### 3.7. Correlation Between Post-Partum Depression and PTSD in Post-Partum Women

**Figure 4:**
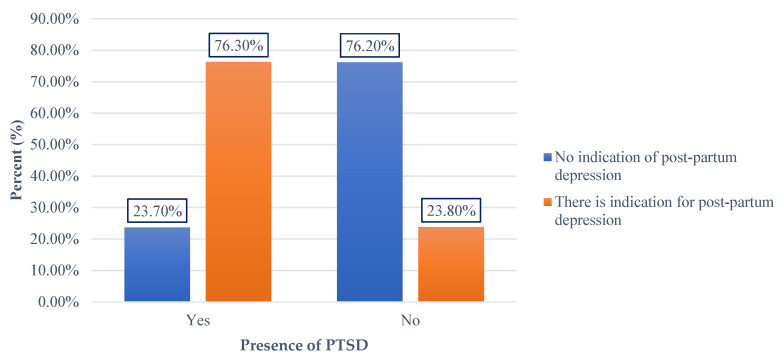
Relationship between post-traumatic stress and the occurrence of post-partum depression.

The analysis of the relationship between post-partum depression and PTSD revealed significant findings. Among the 59 women who experienced PTSD after childbirth, 76.3% (*n* = 45) met the criteria for a diagnosis of post-partum depression. In contrast, only 23.8% (*n* = 66) of the 277 women who did not experience PTSD after childbirth met the criteria for post-partum depression. The x^2^ independence test indicated a statistically significant association between the occurrence of post-traumatic stress following childbirth and post-partum depression, with the results showing x^2^ (1) = 60.473 and *p* = 0.000. These findings demonstrate a clear statistical relationship between post-traumatic stress and post-partum depression (Figure 4).

### 3.8. Correlation Between Post-Partum Depression and Sexual Function in Post-Partum Women

**Figure 5:**
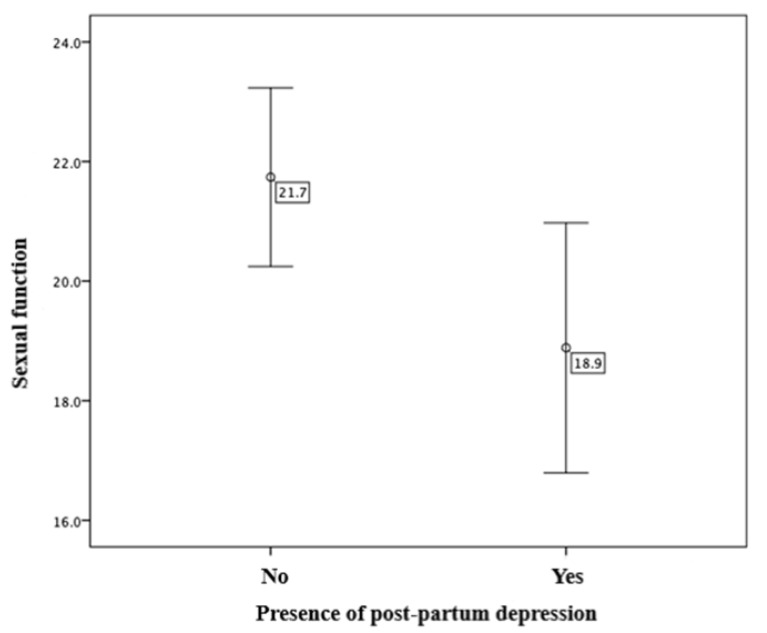
Comparison of sexual functioning in relation to the presence of signs of post-partum depression.

Then, an analysis was conducted to determine whether post-partum depression affects women’s sexual functioning. The average sexual functioning score for women without post-partum depression is 21.7 (SD = 11.4), while for those with post-partum depression, the average score is 18.9 (SD = 11.1). A *t*-test for independent samples revealed a statistically significant difference in average sexual functioning between the two groups, with the results showing t (334) = 2.181 and *p* = 0.030. In conclusion, the presence of post-partum depression is associated with a significantly lower level of sexual functioning in women (Figure 5).

### 3.9. Correlation Between PTSD and Sexual Function in Post-Partum Women

**Figure 6:**
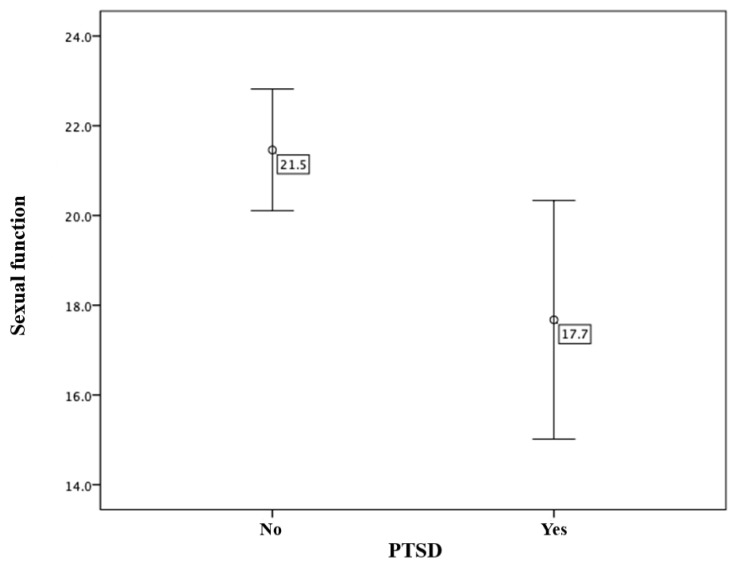
Comparison of sexual functioning in relation to the presence of PTSD.

Lastly, an analysis was conducted to determine whether the presence of PTSD in women who have recently given birth affects their sexual functioning. The results revealed that the average level of sexual functioning for women who did not meet the criteria for PTSD was 21.5 (SD = 11.5), while the average level for those who did meet the criteria was 17.7 (SD = 10.0). A *t*-test for independent samples indicated a statistically significant difference in sexual functioning between the two groups, with the results showing t (334) = 2.344, *p* = 0.020. These results indicate that the presence of post-traumatic stress disorder after childbirth appears to be significantly associated with a decreased level of sexual functioning in women (Figure 6).

## 4. Discussion

Motherhood is a beautiful yet challenging phase in a woman’s life. The total duration of forty weeks of pregnancy, childbirth, the post-partum period, and breastfeeding can directly impact physiological and psychological functions, which may, unfortunately, affect a woman’s sexuality negatively. This study aimed to evaluate the presence of emotional disorders and the level of sexual function observed after childbirth, as well as how sexual function impacts post-partum depression and PTSD and vice versa. We also examined the potential risk factors that may lead to PTSD and post-partum depression.

Our study, based on the results from the EPDS, revealed that the post-partum women who participated had an average score of 10.2 (SD = 6.2). Notably, 33% of these women scored above 14 points, which is considered a borderline value for the prediction of post-partum depression. Additionally, the total score from the FSFI indicated that their level of sexual functioning was moderate, as the women scored an average of 20.8 (SD = 11.3) points on the FSFI, with lower scores on the arousal and orgasm dimensions and higher scores on the satisfaction and pain dimensions [20]. Notably, a correlation between the occurrence of post-partum depression and decreased sexual function during the post-partum period was noticed. From a neurobiological standpoint, depression affects brain regions that are also involved in sexual desire and arousal, such as the prefrontal cortex, amygdala, hypothalamus, and nucleus accumbens. The dysregulation of these areas, especially the reward and motivation circuits, can lead to blunted sexual desire, diminished arousal, and difficulty achieving satisfaction. The chronic activation of the HPA axis and elevated cortisol levels in depression may suppress dopaminergic signaling and oxytocin release, both of which are critical for emotional bonding and sexual pleasure. Temporal dynamics further complicate this relationship [35,36,37]. Post-partum depression can appear within weeks after childbirth, but its effects on sexual functioning may evolve gradually, often persisting for months. Conversely, persistent sexual dissatisfaction may reinforce depressive symptoms, creating a cyclical pattern of emotional and sexual disengagement [38]. This chronic interplay not only impairs relationship satisfaction but also delays psychological recovery, underscoring the importance of early screening and integrative post-partum care that includes sexual health as a core component.

Sexual dysfunction is common among women experiencing depressive symptoms both during pregnancy and after childbirth. Research shows that post-partum depression is linked to a decrease in the frequency and interest in sexual intercourse at 8 to 12 weeks after delivery, as well as a decline in sexual desire at 6 months post-partum [4]. In alignment with our results, in a prospective study conducted in Turkey, in which post-partum depression symptoms were assessed with the EPDS and sexual function with the Index of Female Sexual Function (IFSF), in a sample of 530 women, 2 to 12 months post-partum, a moderate negative correlation (r = −0.60) was found between sexual function and post-partum depression. In the same study, 74.3% of the post-partum women reported experiencing sexual dysfunction, primarily due to factors such as vaginal dryness, prolonged insomnia, fatigue, and lack of time [39]. Dağli et al. [40] conducted a cross-sectional observational study that found similar results regarding the prevalence of female sexual dysfunction during the post-partum period and its relationship with post-partum depression. Their analysis showed a strong, negative, statistically significant correlation between the FSFI and EPDS scores (r = −0.831, *p* = 0.000). According to these findings, it is clear that research indicates a strong association between the risk of post-partum depression and an increased prevalence of sexual dysfunction. These findings are crucial for understanding and addressing the complex interplay between mental health and sexual function in the post-partum period. Moreover, some studies suggest that depression serves as an independent risk factor for sexual dysfunction, with the frequency of dysfunction rising alongside the severity of depression. Overall, other studies support these findings, showing that the presence, duration, and intensity of post-partum depression symptoms are directly related to decreased sexual function following childbirth [41].

Furthermore, our study found that several risk factors are positively linked to a higher likelihood of developing post-partum depression after childbirth. These factors include being over the age of 40, having a low educational level, lacking support from a partner or family, breastfeeding, and experiencing intense traumatic events during the perinatal period. Additionally, research suggests that a mother’s occupation, marital status, availability of social support, socioeconomic status, and financial pressures all play a significant role in the prevalence of post-partum depression following childbirth [42]. In our study, post-partum depression was associated with the level of sexual function during the puerperium. Dawson et al. [16], found a positive correlation between post-partum depression symptoms, lower relationship satisfaction, and sexual problems during the post-partum period. This association was particularly notable among women with a history of sexual dysfunction. In agreement, Khajehei and Doherty [43], in a study that aimed to investigate post-partum depression and sexual dysfunction in Australian women, noticed that symptoms of post-partum depression were found to be significantly linked to several factors: low educational level, exclusive breastfeeding, clinically diagnosed depression, sexual dysfunction, not initiating sexual activity during partnered encounters, and dissatisfaction in relationships. Their study also showed that those with lower educational levels had a 2.2 times higher risk of experiencing depression symptoms. Similarly, the risk increased to 2.5 times for women experiencing sexual dysfunction and to 3.7 times for those who reported dissatisfaction in their relationships. Koçoğlu et al. [44] discovered a negative correlation between the City Birth Trauma Scale, the EPDS, and the FSFI in a study involving 147 post-partum women. Their findings indicated that scores on the City Birth Trauma Scale did not significantly influence the risk of sexual dysfunction. However, higher scores on the EPDS were associated with an increased likelihood of experiencing sexual dysfunction.

The results of our study showed that the existence of post-traumatic stress after childbirth is associated with reduced levels of sexual functioning during the post-partum period. More specifically, it was observed that 17.6% of the post-partum women experience post-traumatic stress after childbirth and meet all four criteria of the PCL scale. Neurobehaviorally, PTSD is characterized by the hyperactivation of the amygdala, dysfunction of the medial prefrontal cortex, and hippocampal volume reduction [45], which together impair emotional regulation, increase vigilance, and contribute to avoidance behaviors. These changes can profoundly interfere with sexual desire and arousal by triggering anxiety, flashbacks, or dissociation during intimate moments. The body’s autonomic responses normally activated during sexual arousal may instead trigger fear or discomfort in women with trauma histories, resulting in emotional detachment, numbing, or sexual aversion. The temporal aspect is again crucial [46,47,48]. Post-traumatic symptoms may not surface immediately but can worsen over time, especially if the traumatic birth experience remains unaddressed. Likewise, the resulting sexual dysfunction may not only be a symptom but also a reinforcer of trauma, especially if women feel ashamed, unsupported, or invalidated in their experiences. This long-term cycle of trauma and sexual distress can significantly impair maternal identity, partner intimacy, and overall mental health [49].

For many years, scientists regarded the experience of giving birth as a positive one for mothers. However, recent research focusing on women who have had traumatic birth experiences has shown that some of these experiences can lead to the development of PTSD. Studies indicate that more than a third of women perceive childbirth as a traumatic experience, and around a quarter of them are believed to develop PTSD following childbirth [19,50]. Post-partum PTSD can have negative effects on the relationship with a partner, including negative emotions and poor communication, reduced intimacy, sexual dysfunction, or even lead to separation [51]. There are reports in which women with birth trauma have described their relationships with their partners as “shattered/broken”. A Norwegian cohort study of 1.480 post-partum women found that women with PTSD two years after giving birth had reduced levels of relationship satisfaction [52]. It has also been found that the presence of PTSD during the post-partum period has a significant impact on the sexual relationships of the parents since intimacy and sexual activity are significantly reduced. More specifically, there are reports of episodes of recall of the birth trauma in the initial phase of sex and reports of avoiding sexual intercourse due to the fear of getting pregnant and giving birth again [53]. Our study revealed that PTSD was significantly correlated with the level of sexual function and post-partum depression.

Although little is known about the impact of post-traumatic stress on couples’ sexuality and the sexual functioning of post-partum women, many studies indicate several risk factors for the development of PTSD immediately after childbirth. According to the literature, depression and/or anxiety during pregnancy are considered the most common psychological factors that can lead to the development of post-partum PTSD [54]. A lack of support from the social environment is the most critical social risk factor for developing PTSD during pregnancy, childbirth, and the post-partum period [55]. The findings of our study showed that risk factors for developing PTSD after childbirth included having an emotional disorder in the past for which they did not receive treatment, lack of support from their partner and family environment, low annual income, negative body image, personal and family history of emotional disorder and early term of baby born. A retrospective study conducted in the Netherlands between 2005 and 2016 examined women who self-reported at least one traumatic birth experience. The main goal was to identify and develop a predictive model for risk factors associated with the development of PTSD during the post-partum period. The study analyzed a sample of 1599 women and found that four significant predisposing factors were related to this risk: a lack of social support, a low sense of coherence, experiences of “threatened death”, and encounters with “previous or imminent injury to the baby” [55]. Similarly, in a longitudinal prospective study conducted in Canada, a history of sexual abuse and higher anxiety sensitivity were found to be determinants of the development of PTSD [56]. In the study by Harris et al. [12], mothers of both preterm and term infants faced significant mental health challenges. Upon hospital discharge, 70% of mothers with very preterm infants and 60% with term infants reported psychological issues. Mothers of very preterm infants showed higher depression levels and PTSD symptoms, while those with term infants primarily experienced increased anxiety. Moreover, research indicates that mothers of very premature infants report significantly higher levels of anxiety, depression, and post-partum stress compared to mothers of full-term infants [57]. Based on the abovementioned, symptoms of depression, anxiety, and stress that women may experience from conception to one year after childbirth can lead to PTSD. Additionally, these symptoms can contribute to sexual dysfunction and a decrease in sexual desire. While PTSD and the poor psychological state during this period may be correlated to physiological changes, research indicates that support from partners and the broader social environment, as well as the quality of social and partner relationships, play a significant role in the variability of depressive and anxiety symptoms, as well as stress in young mothers [58,59].

Furthermore, research shows that while expectant mothers often have a solid social support network, the quality of their relationship with their partner is a crucial protective factor during the perinatal period. Supportive behavior from a partner enhances relationship satisfaction and positively impacts mental and physical health. Additionally, satisfaction and intimacy in the relationship are strong predictors of mental and sexual health during the post-partum period [58,60]. Research indicates that social support plays a crucial role in the emotional and mental health of post-partum women, which also impacts their sexual health. Our study showed that women who lacked a supportive family environment were at an increased risk for developing PTSD and post-partum depression, along with significantly lower levels of sexual functioning. Conversely, women who had a fully supportive family environment experienced the lowest risk of developing PTSD and post-partum depression, as well as higher levels of sexual functioning. In addition to support from the family during the post-partum period, some studies indicate that sharing experiences with other women going through similar situations, such as in maternity support groups and breastfeeding groups, can enhance mental peace and emotional well-being [61].

In summary, this study has several strengths and limitations. It represents a pioneering effort in Greece to evaluate sexual function, post-partum depression, and PTSD after childbirth. Additionally, it examines the correlations between post-partum depression, sexual function, and PTSD, as well as the risk factors for emotional disorders. By providing new insights into the topic of emotional disorders and sexual function during the puerperium period, this study breaks new ground. Secondly, it identifies statistically significant correlations between post-partum depression and PTSD, post-partum depression and sexual function, and PTSD and sexual function, further enhancing our understanding of these conditions. Thirdly, the risk factors associated with PTSD and post-partum depression were found. Considering limitations, one limitation of our study is the lack of evaluations during each 12-month puerperium period, which could provide more comprehensive insights into the long-term trajectory of post-partum emotional and sexual health. Additionally, the recruitment method through online motherhood forums may introduce selection bias, as these platforms are more likely to engage individuals from higher-income or tech-savvy populations. Consequently, this could lead to the underrepresentation of lower-income or tech-limited groups, potentially limiting the generalizability of our findings to broader, more diverse populations.

## 5. Conclusions

Greek post-partum women often face a range of negative emotions, depressive symptoms, and high levels of post-traumatic stress starting from the first month after childbirth. These psychological challenges are significantly associated with the onset of sexual dysfunction, as was observed in our study, yet they are frequently overlooked. Many women struggle silently, often receiving little to no support due to critical or unsupportive family and social environments. This discouragement, coupled with uncertainty about where to seek help, can lead to delayed or absent intervention—ultimately increasing the risk of post-partum depression and PTSD. Given the scarcity of related studies in Greece and the cultural barriers that may prevent open discussion and participation, there is a pressing need for further research. Future studies shoud not only assess the prevalence of mood disorders and sexual difficulties after childbirth but also develop and evaluate personalized approaches for emotional and sexual health support. Additionally, educating gynecological and obstetric care professionals to inform better and support post-partum women may encourage earlier identification and intervention for psychological and sexual health concerns.

## Figures and Tables

**Table 1:** Results of the descriptive and reliability analysis for the 10 questions on the EPDS.

	M	SD	Reliability via Cronbach’s Alpha
I have been able to laugh and see the funny side of things ^b^	0.5	0.8	0.753
I have looked forward with enjoyment to things ^b^	0.8	0.8	0.753
I blamed myself unnecessarily when things went wrong ^a^	1.2	0.9	0.746
I have been anxious or worried for no good reason ^c^	1.8	1.0	0.742
I have felt scared or panicky for no good reason ^c^	1.2	1.1	0.738
I was unable to fulfill my obligations ^a^	1.4	0.8	0.754
I have been so unhappy that I have had difficulty sleeping ^a^	1.0	1.0	0.738
I have felt sad or miserable ^a^	1.1	1.0	0.734
I have been so unhappy that I have been crying ^a^	0.9	0.9	0.742
The thought of harming myself has occurred to me ^c^	0.3	0.6	0.758
Total average score of EPDS	10.2	6.2	0.876

Note: M: mean; SD: standard deviation; a. 0 = Not at all, 3 = Yes, most of the time; b. 0 = As much as I could always, 3 = Not at all; c. 0 = Not at all/Never, 3 = Yes, very often/Yes quite often.

**Table 2:** Multiple regression analysis with the total score of the EPDS as a dependent variable.

	Unstandardized Coefficients		Standardized Coefficients	*t*-Test	*p*-Value	95.0% Confidence Interval for B	
	B	Std. Error	Beta			Lower Bound	Upper Bound
(Constant)	21.381	3.571		5.988	<0.001	14.355	28.406
Age group (years)	−0.227	0.313	−0.037	−0.726	0.468	−0.842	0.388
Place residence	0.025	0.451	0.003	0.055	0.956	−0.862	0.912
Educational level	−0.067	0.335	−0.011	−0.200	0.842	−0.727	0.593
Annual family income	−0.594	0.263	−0.122	−2.260	0.025 *	−1.111	−0.077
Employment status	−0.463	0.531	−0.069	−0.872	0.384	−1.509	0.582
Type of employment	0.189	0.448	0.032	0.422	0.674	−0.693	1.070
Marital status	−0.248	0.967	−0.012	−0.256	0.798	−2.150	1.654
Smoking	−0.383	0.673	−0.029	−0.570	0.569	−1.707	0.940
Drinking	0.332	0.619	0.025	0.537	0.592	−0.885	1.550
Physical activity	−0.333	0.329	−0.048	−1.011	0.313	−0.980	0.315
Body image	−1.861	0.315	−0.289	−5.910	<0.001 *	−2.480	−1.241
Partner support	−1.431	0.447	−0.174	−3.202	0.001 *	−2.311	−0.552
Family support	−0.969	0.429	−0.125	−2.262	0.024 *	−1.813	−0.126
Time of labor before the study enrollment	−0.043	0.235	−0.009	−0.182	0.856	−0.505	0.420
First child	0.743	0.663	0.059	1.121	0.263	−0.562	2.047
Type of delivery	−0.146	0.247	−0.031	−0.589	0.556	−0.633	0.341
Term of baby born	−0.320	0.363	−0.043	−0.882	0.378	−1.035	0.394
Birth expectations	0.571	0.261	0.111	2.186	0.030 *	0.057	1.084
Presence of intense traumatic experience in the perinatal period	1.718	0.735	0.120	2.337	0.020 *	0.271	3.165
Breastfeeding	0.074	0.334	0.011	0.220	0.826	−0.584	0.731
Presence of a medical condition that requires treatment	−0.298	0.720	−0.019	−0.413	0.680	−1.715	1.119
Presence of personal emotional disorder in the past	2.998	0.481	0.315	6.237	<0.001 *	2.052	3.944
Presence of a family history of any emotional disorders	−0.542	0.634	−0.041	−0.854	0.394	−1.789	0.706
R^2^ = 0.362, F = 7.694, *p* < 0.001							

Note: * statistically significant at *p* < 0.05.

**Table 3:** Descriptive and reliability analysis results for the 20 questions of the PCL scale.

	M	SD	Reliability via Cronbach’s Alpha
**Criteria B**	0.6	0.9	0.728
Repeated, disturbing, and unwanted memories of the stressful experience?	0.9	1.3	0.745
Repeated, disturbing dreams of the stressful experience?	0.4	0.8	0.751
Suddenly feeling or acting as if the stressful experience were actually happening again (as if you were actually back there reliving it)?	0.4	0.9	0.749
Feeling very upset when something reminded you of the stressful experience?	0.9	1.2	0.744
Having strong physical reactions when something reminded you of stressful experiences (for example, heart pounding, trouble breathing, sweating)?	0.6	1.0	0.747
**Criteria C**	0.9	0.8	0.747
Avoiding memories, thoughts, or feelings related to the stressful experience?	1.0	1.3	0.746
Avoiding external reminders of stressful experiences (for example, people, places, conversations, activities, objects, or situations)?	0.9	1.3	0.745
**Criteria D**	0.9	0.9	0.791
Trouble remembering important parts of the stressful experience?	0.5	1.0	0.750
Having strong negative beliefs about yourself, other people, or the world (for example, having thoughts such as: I am bad, there is something seriously wrong with me, no one can be trusted, the world is completely dangerous)?	1.0	1.2	0.744
Blaming yourself or someone else for the stressful experience or what happened after it?	0.9	1.3	0.744
Having strong negative feelings such as fear, horror, anger, guilt, or shame?	0.8	1.2	0.746
Loss of interest in activities that you used to enjoy?	1.3	1.3	0.746
Feeling distant or cut off from other people?	1.2	1.3	0.744
Trouble experiencing positive feelings (for example, being unable to feel happiness or have loving feelings for people close to you)?	1.0	1.3	0.745
**Criteria E**	1.0	0.8	0.770
Irritable behavior, angry outbursts, or acting aggressively?	1.6	1.4	0.745
Taking too many risks or doing things that could cause you harm?	0.2	0.6	0.756
Being “superalert” or watchful or on guard?	1.2	1.3	0.746
Feeling jumpy or easily startled?	0.7	1.1	0.749
Having difficulty concentrating?	1.2	1.2	0.746
Trouble falling or staying asleep?	1.1	1.3	0.748

Note: M: mean; SD: standard deviation.

**Table 4:** Results of descriptive analysis for the percentage of women meeting criteria for each of the four criteria for post-traumatic stress disorder.

	No		Yes	
	*n*	%	*n*	%
**Criteria B**	221	65.8%	115	34.2%
**Criteria C**	224	66.7%	112	33.3%
**Criteria D**	191	56.8%	145	43.2%
**Criteria E**	196	58.3%	140	41.7%

Note: Data are expressed as frequencies (*n*) and percentages (%).

**Table 5:** Multiple regression analysis with a total score of PTSD, based on the PCL-5, as a dependent variable.

	Unstandardized Coefficients		Standardized Coefficients	*t*-Test	*p*-Value	95.0% Confidence Interval for B	
	B	Std. Error	Beta			Lower Bound	Upper Bound
(Constant)	60.672	8.993		6.746	<0.001	42.977	78.367
Age group (years)	−1.251	0.767	−0.081	−1.632	0.104	−2.759	0.257
Place residence	−0.0957	1.107	−0.040	−0.864	0.388	−3.136	1.222
Educational level	0.770	0.837	0.050	0.920	0.358	−0.877	2.416
Annual family income	1.478	0.649	0.112	2.275	0.024 *	0.200	2.755
Employment status	0.208	1.308	0.012	0.159	0.873	−2.364	2.781
Type of employment	0.197	1.106	0.013	0.178	0.859	−1.979	2.373
Marital status	−1.207	2.404	−0.023	−0.502	0.616	−5.937	3.524
Smoking	−1.249	0.656	−0.100	−1.903	0.058	−2.540	0.042
Drinking	−1.918	1.531	−0.057	−1.253	0.211	−4.930	1.093
Physical activity	−0.003	0.817	0.000	−0.003	0.997	−1.610	1.605
Body image	−3.047	0.795	−0.185	−3.831	<0.001 *	−4.611	−1.482
Partner support	−3.131	1.157	−0.134	−2.706	0.007 *	−5.407	−0.855
Family support	−2.919	1.027	−0.141	−2.844	0.005 *	−4.939	−0.899
Time of labor before the study enrollment	−0.107	0.584	−0.009	−0.183	0.855	−1.256	1.042
First child	0.818	1.651	0.026	0.496	0.620	−2.430	4.067
Type of delivery	−1.232	0.614	−0.102	−2.006	0.046 *	−2.440	−0.023
Term of baby born	−0.079	0.022	−0.198	−3.593	<0.001 *	−0.122	−0.036
Birth expectations	4.742	1.839	0.129	2.578	0.010 *	1.123	8.361
Presence of intense traumatic experience in the perinatal period	0.157	0.033	0.267	4.768	<0.001 *	0.092	0.222
Breastfeeding	0.068	0.842	0.004	0.081	0.936	−1.588	1.725
Presence of a medical condition that requires treatment	0.099	1.577	0.003	0.062	0.950	−3.005	3.202
Presence of personal emotional disorder in the past	8.138	1.184	0.333	6.875	<0.001 *	5.809	10.467
Presence of a family history of any emotional disorders	−0.053	0.044	−0.065	−1.211	0.227	−0.140	0.033
R^2^ = 0.399, F = 8.995, *p* < 0.001							

Note: * statistically significant at *p* < 0.05.

**Table 6:** Descriptive and reliability analysis results of post-partum women’s sexual functioning subscales and overall levels.

	M	SD	Min	Max	Reliability via Cronbach’s Alpha
Desire	3.4	1.5	1.2	6.0	0.808
Arousal	3.1	2.2	0.0	6.0	0.772
Lubrication	3.5	2.4	0.0	6.0	0.769
Orgasm	3.2	2.4	0.0	6.0	0.769
Satisfaction	3.9	1.9	0.8	6.0	0.787
Pain	3.6	2.4	0.0	6.0	0.774
**Total Sexual function**	20.8	11.3	2.0	36.0	0.944

Note: Μ: mean; SD: standard deviation; Min: minimum value; Max: maximum value.

## Data Availability

The data presented in this study are available upon request from the corresponding author. The data are not publicly available due to ethical restrictions.

## Supplementary Materials

The following supporting information can be downloaded at https://www.mdpi.com/article/10.3390/diagnostics15091065/s1, Table S1: Demographic, lifestyle, and body image data of post-partum women (N = 336); Table S2: Data on support from spouse/partner and support from the family environment; Table S3: Data about childbirth; Table S4: Health status and emotional disorder history.
