# Supplementary material for: Emotional Disorders, Risk Factors, and Correlations of Post-Partum Depression and Post-Traumatic Stress Disorder with Sexual Function During Post-Partum Period
Source: Diagnostics (Basel). 2025 Apr 22;15(9):1065. doi: 10.3390/diagnostics15091065 (PMC12071330; doi:10.3390/diagnostics15091065)
Supplement: Supplementary file 1 [file diagnostics-15-01065-s001.zip › diagnostics-3537836-supplementary.pdf]

diagnostics

MDPI

Supplementary Materials

Table S1. Demographic, lifestyle and body image data of postpartum women (N=336).

**n (%)**			
**Age group (years)**	a)	18-25	33 (9.8%)
	b)	26-30	96 (28.6%)
	c)	31-35	124 (36.9%)
	d)	36-40	65 (19.3%)
	e)	Over 40	18 (5.4%)
**Place of residence**	a)	Urban area	189 (56.2%)
	b)	Suburban area	114 (33.9%)
	c)	Rural area	33 (9.8)%
**Educational level**	a)	No education/unfinished school	7 (2.1%)
	b)	High school diploma	70 (20.8%)
	c)	Vocational Training Institute diploma	79 (23.5%)
	d)	Bachelor's degree	133 (39.6%)
	e)	Master's degree	47 (14.0%
**Annual family income**	a)	Up to 5000 €	52 (15.5%)
	b)	5001-10000 €	76 (22.6%)
	c)	10001-20000 €	129 (38.4%)
	d)	20001-30000 €	46 (13.7%)
	e)	30001-40000	16 (4.8%)
	f)	Over 40000	17 (5.1%)
**Employment status**	a)	Not working	141 (42.0%)
	b)	Part-time working	52 (15.5%)
	c)	Full-time working	143 (42.6%)
**Type of employment**	a)	Housewife	123 (36.6%)
	b)	Self-employed	43 (12.8%)
	c)	Private employ	137 (40.8%)
	d)	Public employ	33 (9.8%)
**Marital status**	a)	Single	3 (0.9%)
	b)	In a relationship / Engaged	14 (4.2%)
	c)	Married	313 (93.2%)
	d)	Divorced	6 (1.8%)

**Smoking**	a) No, I never smoked	151 (44.9%)
	b) No, I cut it because of the child	67 (19.9%)
	c) Yes, occasionally	46 (13.7%)
	d) Yes, systematically	72 (21.4%)
**Drinking**	a) Rarely, less than 1 time per week	298 (88.7%)
	b) A little, 1-2 times per week	30 (8.9%)
	c) Moderately, 3-4 times per week	4 (1.2%)
	d) Quite often, almost daily	4 (1.2%)
**Physical Activity**	a) No, I don't have time	201 (59.8%)
	b) No, I don't like sports	62 (18.5%)
	c) Yes, when I have time	61 (18.2%)
	d) Yes, systematically	12 (3.6%)
**Body image**	a) Negatively, I look at myself and I get disappointed.	79 (23.5%)
	b) Moderately negative	86 (25.6%)
	c) Moderately positive	136 (40.5%)
	d) Positively, I really like myself!	35 (10.4%)

Note: Data are expressed as frequencies (n) and percentages (%).

Table S2. Data on support from spouse/partner and support from family environment.

**n (%)**		
**The other half, after birth, is for the woman.**	a) Abusive	1 (0.3%)
	b) Not at all supportive	5 (1.5%)
	c) Slightly supportive	15 (4.5%)
	d) Less supportive than she would like	87 (25.9%)
	e) Completely supportive	228 (67.9%)
**The family environment, after birth, is for the woman**	a) Not at all supportive	9 (2.7%)
	b) Slightly supportive	29 (8.6%)
	c) Less supportive than she would like	85 (25.3%)
	d) Completely supportive	213 (63.4%)

Note: Data are expressed as frequencies (n) and percentages (%).

Table S3. Data about childbirth.

		n (%)
**Time of labor before the study enrollment**	a) Less than a month	25 (7.4%)
	b) 1-2 months	22 (6.5%)
	c) Over 2 and less than 4 months	54 (16.1%)
	d) Over 4 and less than 6 months	60 (17.9%)
	e) Over 6-12 months	175 (52.1%)
**First child**	a) No	139 (41.4%)
	b) Yes	197 (58.6%)
**Type of delivery**	a) Planned C-section	101 (30.1%)
	b) Emergency C-section	58 (17.3%)
	c) Normal	121 (36.0%)
	d) Vacuum Extraction Delivery	13 (3.9%)
	e) She tried naturally but had a caesarean section	43 (12.8%)
**The baby was born**	a) Early term, required prolonged hospitalization in the Neonatal Unit	10 (3.0%)
	b) Early term but did not require hospitalization in the Neonatal Unit	34 (10.1%)
	c) full term, but required hospitalization in the Neonatal Unit	13 (3.9%)
	d) Full term	265 (78.9%)
	e) Early term, required brief hospitalization in Neonatal Unit	14 (4.2%)
**Did the birth experience meet your expectations about it?**	a) No, the way I gave birth was a traumatic experience for me.	95 (28.3%)
	b) Yes, it was a wonderful experience.	241 (71.7%)
**Intense traumatic experience in the perinatal period**	a) No	254 (75.6%)
	b) Yes	82 (24.4%)
**Breastfeeding**	a) I didn’t breastfeed by choice	33 (9.8%)
	b) I wanted to breastfeed, but I didn’t have milk	30 (8.9%)
	c) I breastfed for a while	126 (37.5%)
	d) I’m still breastfeeding	147 (43.8%)

Note: Data are expressed as frequencies (n) and percentages (%).

Table S4. Health Status and Emotional Disorder History.

**n (%)**		
**Do you have a medical condition that requires treatment?**	a) No	268 (79.8%)
	b) Yes	68 (20.2%)
**Have you had any emotional disorders in the past? (e.g., depression, anxiety disorder, etc.)**	a) No	234 (69.6%)
	b) Yes, but I didn't need treatment.	72 (21.4%)
	c) Yes, and I received treatment.	30 (8.9%)
**Is there a family history of any emotional disorders, such as depression, anxiety disorders, or bipolar disorder?**	a) No	228 (67.9%)
	b) Yes	108 (32.1%)

Note: Data are expressed as frequencies (n) and percentages (%).
